# Micro-invasive glaucoma surgery – an interventional glaucoma revolution

**DOI:** 10.1186/s40662-019-0154-1

**Published:** 2019-09-29

**Authors:** Manjool Shah

**Affiliations:** Department of Ophthalmology and Visual Sciences, Kellogg Eye Center, University of Michigan, 1000 Wall Street, Ann Arbor, MI 48105 USA

**Keywords:** Micro-invasive glaucoma surgery, Glaucoma, Individualized care, Glaucoma surgery

## Abstract

The glaucoma surgical landscape has changed dramatically over the last decade with the introduction and integration of micro-invasive glaucoma surgery (MIGS) techniques. These modalities target physiologic outflow pathways or optimize previously utilized glaucoma surgical methods in order to deliver safety, efficacy, and individualized care to the patient. MIGS techniques can be classified based on anatomical location as well as method of intraocular pressure (IOP) reduction. This review will focus on MIGS optimizing the conventional outflow pathway via intervention at Schlemm’s canal, MIGS optimizing the uveoscleral outflow pathway via suprachoroidal shunting, and MIGS optimizing the transscleral or subconjunctival outflow pathway which has long been utilized by glaucoma surgeons performing traditional filtration procedures. The wide array of currently available MIGS modalities can be staggering to the glaucoma care provider, but an understanding of the landscape and the large classes of interventional strategies can allow for clinical decision making based on the specifics of the patient’s needs and the pathophysiology of their disease.

## Background

Glaucoma is well recognized as a leading cause of global vision loss and blindness, with over 100 million people expected to suffer from the disease by 2040 [1]. Conventional strategies to control glaucoma focus on reduction of intraocular pressure (IOP). The mainstay of glaucoma therapy is pharmaceutical, with the utilization of various classes of topically applied ocular hypotensive agents. While the number of pharmaceutical agents has steadily increased over the last several decades, fundamental challenges to medical therapy continue to exist. Cost, local and systemic adverse effects, and adherence remain impediments to success with topical medical therapy. Laser trabeculoplasty has also emerged as a useful adjunct to conventional medical therapy, and recent studies have suggested that laser trabeculoplasty may be at least as effective as medical therapy early in the management of glaucoma [2]. Unfortunately, the combination of medical and laser-based therapies may not be enough to control the IOP of all glaucoma patients. As such, surgical intervention has long been an integral part of the algorithm of care. The standard surgical paradigm involves bypassing the eye’s natural aqueous outflow pathways by creating external outflow into the subconjunctival space. The main methods of doing so are trabeculectomy or the glaucoma drainage device. Both surgical modalities have demonstrated efficacy in numerous clinical trials [3]. However, these surgical strategies carry a significant risk of vision-threatening morbidity to the patient. In the Primary Tube Versus Trabeculectomy (PTVT) Study, serious complications resulting in loss of vision or need for reoperation occurred in 1% of the drainage device group and 7% of the trabeculectomy group [3]. While such surgical methods certainly have their role in the treatment armamentarium, the relatively high morbidity does not justify their use in all patients for whom medical and laser-based strategies have failed.

The era of micro-invasive glaucoma surgery (MIGS), began as technology evolved, demographic pressures increased, and the glaucoma care community recognized that a new interventional strategy needed to exist to take care of patients who required more IOP control than can be provided by medical and laser-based approaches but who do not need aggressive surgical intervention. The traditional tenets of MIGS are that they are delivered through an ab-interno, micro-incisional approach, demonstrate moderate efficacy, are minimally traumatic, emphasize safety, and ensure a rapid recovery for the patient [4]. Over the last decade, the MIGS space has grown tremendously, and has become a major part of the glaucoma surgical paradigm. Fundamentally, this growth has enabled glaucoma care providers to provide more nuanced, patient-centric care.

This review will focus on several of the currently available MIGS strategies and devices. In order to logically approach this space, it is important to classify the various approaches based on the site of anatomical intervention and augmentation. The rationale for classification is based on physiological principles that can govern efficacy and safety of a given family of techniques. As such, this review will categorize MIGS strategies as Schlemm’s canal MIGS, suprachoroidal MIGS, subconjunctival MIGS, and novel surgical targets.

## Main text

### Schlemm’s canal MIGS

The family of micro-invasive strategies directed at Schlemm’s canal and the conventional outflow system is likely the richest of the MIGS groups. The pathophysiologic rationale for intervening at this anatomical location lies in bypassing the resistance to aqueous outflow imparted by the trabecular meshwork tissue. By bypassing such resistance, there exists a theoretical possibility of achieving an IOP that is similar to the episcleral venous pressure. Various methods have been developed to achieve bypass of the trabecular meshwork, namely microstenting, micro-incisions, and viscodilation.

Microstenting strategies have grown over the last decade. The original Schlemm’s canal microstent is the first generation iStent (Glaukos Corp., San Clemente, CA, USA), which was introduced in the United States in 2012. While the original clinical trials demonstrated moderate efficacy when these stents were used in combination with cataract extraction [5, 6], their continued utilization in patients with open angle glaucoma has consistently demonstrated safety and efficacy. This strategy has led Glaukos to develop a second generation iStent, known as the iStent Inject [7], which achieved United States FDA approval in 2018. Additionally, another Schlemm’s canal microstent has been developed by Ivantis, Inc. (Irvine, CA, USA), called the Hydrus Microstent [8].

Fundamentally, all three microstents serve to bypass the resistance of the trabecular meshwork by allowing aqueous humor to directly flow into Schlemm’s canal. However, there are subtle differences between the stents that may be relevant. The original first generation iStent is a single stent system. However, studies [9] have suggested that multiple stents may achieve greater efficacy than a single stent. As such, while the iStent Inject is a smaller individual stent, two stents are included in the system. The difference in lumen diameter and size is likely not relevant with regard to the fluid dynamics of aqueous outflow, but the ability to access a broader area of the conventional outflow system with a second stent may be of value. The Hydrus Microstent takes a different strategy to broadening the area of coverage; instead of utilizing multiple stents, the Hydrus Microstent is in and of itself a longer device. At 8 mm in length, the stent spans three clock hours of Schlemm’s canal. Additionally, the stent provides both a direct bypass of trabecular meshwork and a stretching of trabecular meshwork through its multimodal mechanism of action. In a randomized controlled trial comparing two iStents versus the Hydrus Microstent, while the IOP results were similar between the two groups, the Hydrus cohort required fewer medications and were more likely to be medication free [10]. Additional comparative studies will ultimately be necessary to further validate these findings, and it is reassuring that IOP control can safely be achieved with a variety of microstenting approaches.

Micro-incisional approaches have also grown in popularity over the last several years. Goniotomy and trabeculotomy techniques have long been a mainstay in the surgical management of pediatric and congenital glaucomas. In recent years, recognition of the utility of this surgical approach in adult glaucomas has taken hold. Various strategies exist to incise the trabecular meshwork in order to create a direct pathway for aqueous humor into Schlemm’s canal and beyond. The Kahook Dual Blade (New World Medical, Rancho Cucamonga, CA, USA) and the newer Goniotome (NeoMedix Corp., Tustin, CA, USA) utilize blades on both sides of a footplate that excise a bloc of trabecular tissue by making incisions at the anterior and posterior margins. Fundamentally, both devices are simply tools to create this controlled incision, and as such one would expect similar efficacy between the devices. Numerous reports have shown efficacy similar to other Schlemm’s canal-based MIGS strategies in patients with the entire spectrum of disease severity [11, 12]. Furthermore, in the first comparison between modalities, there was a greater percent reduction in IOP and number of medications in the goniotomy group as compared to the iStent group, although both methods resulted in a similar IOP [13].

As compared to a limited goniotomy using a specialized dual blade, in which tissue is excised 180 degrees from the surgeon, the gonioscopy-assisted transluminal trabeculotomy (GATT) procedure utilizes either a microcatheter or a blunted suture to create a circumferential ab-interno infracture of the trabecular meshwork [14]. Strengths of this circumferential technique include the ability to reach all 360 degrees of the distal outflow system as well as the ability to do so using a potentially cost-effective method [15]. Longer-term retrospective outcome data have documented continued efficacy for the vast majority of patients [16].

In an effort to be even less injurious to the existing anatomical structures of the anterior chamber angle, viscodilation has been utilized to reduce outflow resistance in the trabecular meshwork tissue. By distending and enlarging Schlemm’s canal, adjacent juxtacanalicular trabecular meshwork and distal collector channels, IOP reduction can be achieved with minimal disruption. Current methods to perform viscodilation involve the ab-interno canaloplasty, or AbIC procedure, as well as the Omni procedure (Sight Sciences, Inc., Menlo Park, CA, USA). Early evidence suggests safety and efficacy, again on par with other Schlemm’s canal techniques [17].

Despite a wealth of strategies to optimize conventional outflow through the Schlemm’s canal interventional route, some common points of failure are inevitable. Firstly, there is a recognition of the fact that wound healing can occur in this region, which may result in scarring in the area of microstent implantation or microincision placement. Previous reports have identified scenarios in which such scarring has resulted in increased IOP and potential need for additional intervention [18, 19]. Furthermore, patients with glaucoma may have clinically significant resistance to outflow distal to Schlemm’s canal which may elevate the floor of IOP reduction after intervention. There may be a correlation between disease severity and distal outflow disease, as postulated by Grover et al. and corroborated by findings of distal outflow sclerosis in other studies [16, 20, 21]. While continued investigation and augmentation of technique is inevitable and necessary, the central presence of Schlemm’s canal-based MIGS in the glaucoma treatment algorithm appears to be steadfast.

### Suprachoroidal MIGS

Just as the Schlemm’s canal-based MIGS aim to augment the conventional physiologic outflow pathway, the suprachoroidal MIGS procedures aim to take advantage of the uveoscleral pathway to reduce IOP. Unlike the conventional pathway, uveoscleral outflow is not subject to an IOP floor. As a result, there is theoretically greater IOP-reducing capacity to this system. The negative pressure gradient of the suprachoroidal space is the driver of the uveoscleral pathway [22], and surgical device placement to augment this outflow has the potential to convey a substantial IOP reduction.

The first MIGS device to target this space was the Cypass device (Alcon, Ft. Worth, TX, USA). Early evidence showed significant IOP and medication reduction for this procedure when combined with cataract extraction [23]. However, the Cypass device was ultimately recalled in 2018 when 5-year data suggested a clinically significantly increased rate of corneal endothelial cell loss in certain patients with the device. Specifically, patients in whom the Cypass device was more prominently positioned in the anterior chamber as opposed to deeper in the angle and suprachoroidal space exhibited greater endothelial cell loss. Given that this is a positioning problem that is potentially addressable, the overall strategy of suprachoroidal stenting is not necessarily invalidated.

Other devices for augmenting uveoscleral outflow remain in development and in the investigational pipeline. Glaukos Corporation (San Clemente, CA, USA) is actively investigating the iStent Supra, and iStar Medical (Wavre, Belgium) is investigating the MINIject device. The iStent Supra is a small stent 4 mm in length with a mild curvature to follow the curve of the sclera. In contrast, the MINIject is composed of a novel porous silicone material that allows for controlled aqueous outflow and limited tissue integration.

As this surgical space is relatively sparse, more investigation and clinical data are needed. Nevertheless, there are potential concerns for failure with this surgical target. Specifically, wound healing may result in inadequate IOP reduction. On the other extreme, given the low IOP floor of the suprachoroidal space, there is the theoretical risk of clinically significant hypotony, ciliary effusions, and other choroidal pathology. The unique designs and material properties of the above mentioned investigational devices may mitigate some of these risks, and glaucoma interventionalists hope to have MIGS that access the uveoscleral outflow pathway available to them for certain patients.

### Subconjunctival MIGS

In contrast to the MIGS outflow strategies described above, the subconjunctival route is fundamentally non-physiologic. Aqueous humor does not naturally flow into the subconjunctival space, and any attempt to create a pathway into this space may be met with a scarring response. However, the subconjunctival surgical target has long been a mainstay of conventional glaucoma surgical intervention, with older surgical modalities like trabeculectomy and glaucoma drainage devices employing this route. The rationale behind a MIGS approach to this space primarily focuses on predictability, control, and as a result, safety. By combining already established knowledge regarding subconjunctival and episcleral wound healing with advancements in device fabrication, the subconjunctival space offers a powerful pathway for IOP reduction. Of course, successful subconjunctival outflow will result in the formation of an aqueous bleb.

The methodology for predictability and control with subconjunctival MIGS lies in the properties of fluid dynamics. As described by Hagen and Poiseuille, resistance to outflow is proportional to length and radius of the path of fluid [24]. Taking advantage of these principles allows for a device to have an inbuilt outflow resistance while also allowing early and immediate aqueous flow. As a result, there is a floor to how low IOP can go, thus mitigating the risk of hypotony and associated sequelae.

There are currently two devices that take advantage of these fluid dynamics properties in their design and implementation. The Xen gel stent (Allergan, Inc., Irvine, CA, USA) has a 45 μm diameter internal lumen and is delivered using an ab-interno approach through a corneal incision [25]. When the stent emerges in the subconjunctival space, Hagen-Poiseuille’s law predicts a resistance of approximately 7.5 mmHg, which has been validated experimentally [24]. Ab-interno deployment of this microstent essentially eliminates the need for conjunctival incisions and subsequent closure, and as a result the risk of wound leakage is essentially absent.

An investigational device that also takes advantage of the properties of fluid dynamics to achieve flow restriction and increased safety is the Preserflo device (Santen Co., Japan). While implantation of this microshunt requires an ab-externo delivery via a conjunctival incision and dissection and a scleral tunnel, a theoretical advantage lies in the design and materials utilized in the device. Specifically, the Preserflo device is composed of a material known as SIBS, which has been shown experimentally to be uniquely biocompatible and nonreactive [26]. As episcleral healing and scarring is a major factor in subconjunctival surgical failure, any attempt to mitigate this reaction may be met with success, although results from clinical studies of this device are pending to validate this rationale fully.

### Novel surgical targets

The proliferation of MIGS technologies has allowed for creative approaches to the problem of safe and regulated IOP control. Innovation and development continue to be prevalent, and newer MIGS approaches are to be expected. One example of a unique approach to IOP control is the Beacon Aqueous Microshunt (MicroOptx, Maple Grove, MN, USA). The Beacon device is implanted via a clear corneal incision and allows for aqueous outflow onto the ocular surface. By using proprietary material property and nanoscale fabrication techniques, its developers claim to avoid the risk of pathogen migration into the anterior chamber. As this strategy is completely novel, it is difficult to predict potential benefits or challenges that may accompany it. Clinical trials of this device are being designed.

## Conclusions

The glaucoma surgical space has grown dramatically, and interventional strategies and approaches continue to actively evolve. With so many options available, clinicians may face a form of choice paralysis in selecting the right procedure or approach for a given patient. The next phase in technique evaluation will involve identification of biomarkers based on patient and disease factors to help tailor therapy in an individualized manner. Early work has already begun in this space by recognizing the pathophysiological basis for certain forms of glaucoma. For example, the use of a Schlemm’s canal-based technique has shown marked efficacy in the treatment of steroid-induced glaucoma, which is a disease that primarily affects the trabecular meshwork [27]. Additionally, well-designed randomized clinical trials will need to be developed to better understand the relative strengths and weaknesses of various MIGS approaches within and across the groups delineated above.

Ultimately, removing the challenges of medication adherence and placing the control of IOP in the hands of the glaucoma care provider will be instrumental in advancing management of this disease. With innovation in sustained release pharmacotherapy, the possibility of combined minimally invasive surgical and pharmaceutical intervention by the glaucoma physician is within grasp. The goal of all physicians should be to prioritize the patient’s wellbeing and quality of life; the MIGS revolution has allowed physicians to individualize care and thereby meet this goal. Continued innovation will only broaden access for even more patients in the years to come.

## Data Availability

Not applicable.
